# Risk factors for hematoma in patients undergoing cardiac device procedures: A WRAP-IT trial analysis

**DOI:** 10.1016/j.hroo.2022.05.012

**Published:** 2022-06-16

**Authors:** Khaldoun G. Tarakji, Panagiotis Korantzopoulos, Francois Philippon, Mauro Biffi, Suneet Mittal, Jeanne E. Poole, Charles Kennergren, Daniel R. Lexcen, Jeff D. Lande, Gregory Hilleren, Swathi Seshadri, Bruce L. Wilkoff

**Affiliations:** ∗ Department of Cardiovascular Medicine, Cleveland Clinic, Cleveland, Ohio; † First Department of Cardiology, University Hospital of Ioannina, Ioannina, Greece; ‡ Cardiology Department, Institut Universitaire de Cardiologie et de Pneumologie de Québec (IUCPQ), Quebec, Canada; § Cardiology, Policlinico Sant' Orsola, Malpighi, Italy; ‖ Electrophysiology, Valley Health System, Ridgewood, New Jersey; ¶ Division of Cardiology, University of Washington School of Medicine, Seattle, Washington; # Cardiothoracic Surgery, Sahlgrenska University Hospital, Göteborg, Sweden; ∗∗ Cardiac Rhythm Management, Medtronic, Inc, Mounds View, Minnesota

**Keywords:** CIED, Hematoma, Complication, Risk factor, Antithrombotic management, Anticoagulation, Antiplatelet

## Abstract

**Background:**

Implant site hematoma is a known complication of cardiac device procedures and can lead to major consequences.

**Objectives:**

To evaluate risk factors for hematoma and further understand the relationship between anticoagulant (AC), antiplatelet (AP) use, and hematoma development.

**Methods:**

We included 6800 patients from the WRAP-IT trial. To assess baseline and procedural characteristics associated with hematoma within the first 30 days postprocedure, a stepwise Cox regression model was implemented with minimal Akaike information criterion. Cox regressions were also used to evaluate AC/AP use and hematoma risk.

**Results:**

The overall rate of hematoma was 2.2%. The model identified 11 baseline and procedural characteristics associated with hematoma risk. AC use (hazard ratio [HR]: 2.44, *P* < .001), lower body mass index (HR: 1.06, *P* < .001), and history of valve surgery (HR: 2.11, *P* < .001) were associated with the highest risk. AP use, male sex, history of coronary artery disease, existing pocket, history of nonischemic cardiomyopathy, number of previous cardiac implantable electronic device (CIED) procedures, procedure time, and lead revision were associated with moderate risk. Antithrombotic use was high overall (86%) and AC+AP use was highly predictive of hematoma risk. Regardless of AC status, AP use was associated with an almost doubling of risk vs no AP (HR = 1.85, *P* = .0006) in the general cohort. Interruption of AC was associated with the lowest hematoma risk (HR = 2.35) while heparin bridging (HR = 4.98) and AP use vs no AP use (HR = 1.85) was associated with the highest hematoma risk.

**Conclusion:**

The results of this analysis highlight risk factors associated with the development of hematoma in patients undergoing CIED procedures and can inform antithrombotic management.

**Key Findings**

▪ In this large prospectively followed cohort of patients undergoing secondary CIED procedures or initial CRT-D implantation, the incidence of hematoma within 30 days was 2.2%.
▪ Antithrombotic use, in general, was highly predictive of hematoma risk, and in this global cohort close to 86% of patients were on AC or AP at the time of their CIED procedure.
▪ Varying antithrombotic regimens impacted hematoma risk whereby the use of warfarin was associated with >3× risk, use of DOAC with ∼2× risk, and antiplatelet use with ∼2× risk. AC management strategies also had an influence on hematoma risk, with an interrupted AC strategy being associated with the lowest risk of hematoma (HR = 2.35), while heparin bridging (HR = 4.98) and AP use vs no AP use (HR = 1.85) were associated with a higher risk of hematoma.

## Introduction

The incidence of implant site hematoma after cardiac implantable electronic device (CIED) procedures has been reported to range from 1.2% to 9.5%^1^, ^2^, ^3^, ^4^, ^5^, ^6^, ^7^ and is associated with serious complications including device infection, potential adverse event of cessation of oral anticoagulation therapy, prolonged hospitalization, increased healthcare costs, morbidity, and mortality.^2^^,^^8^^,^^9^ Previously reported risk factors for hematoma include age, history of stroke, congestive heart failure, renal failure, type of anticoagulation therapy, type of device, and operator experience.^2^^,^^3^^,^^5^^,^^7^^,^^10^^,^^11^ However, prospective data are still needed, as there are inconsistencies among prior reports, and most are retrospective in nature. More importantly, with the ubiquitous use of antithrombotics, management of these agents represents a common challenge of weighing the risk of bleeding with continued therapy vs the risk of systemic thromboembolism with interrupted therapy. General guidelines addressing the perioperative management of antithrombotics recommend bridging with heparin products among patients at high risk for systemic thromboembolism; these usually include patients with mechanical valves, recent strokes, or atrial fibrillation with CHA_2_DS_2_-VASc score of 5 or more.^12^ The management of moderate- or low-risk patients is less clear, and these guidelines do not consider the unique characteristics of CIED procedures and the consequences of hematomas.

The use of antithrombotic agents among patients undergoing CIED procedures has been shaped by several randomized clinical trials. The BRUISE CONTROL study^7^ showed that continued warfarin therapy markedly reduced the risk of hematoma compared to bridging with heparin, while BRUISE CONTROL-2^13^ showed no significant difference in the incidence of hematoma between continued vs interrupted direct oral anticoagulants (DOAC) at the time of the procedure. The use of concomitant antiplatelet agents with any anticoagulants, whether warfarin or DOAC, confers an additional risk of hematoma.^14^ Nevertheless, the management of patients on antithrombotic therapy who are at moderate or low thromboembolic risk remains vague and hematoma risk is not well characterized.

The Worldwide Randomized Infection Prevention Trial (WRAP-IT) provides a unique opportunity to assess the real-world influence of patient- and procedure-related risk factors that may be associated with the development of hematoma. WRAP-IT showed a significant reduction in major CIED infection with the use of the TYRX^TM^ absorbable antibacterial envelope (Medtronic, Inc, Minneapolis MN) in patients undergoing replacement/revision/upgrade procedures or initial cardiac resynchronization therapy defibrillator (CRT-D) implants.^15^ The management of antithrombotic agents was left up to the discretion of the operators and sites; however, the type and strategy of antithrombotic use was captured as part of the study along with a number of other detailed procedure characteristics.

In a recent analysis of the WRAP-IT data set, the development of hematoma conferred a greater than 11-fold risk of developing a major CIED infection among patients that did not receive the antibacterial envelope during their index procedure.^9^ The purpose of this analysis is therefore to evaluate patient and procedural risk factors attributed to the occurrence of hematomas and to further understand the relationship between hematoma and antithrombotic use among the WRAP-IT trial patients.

## Methods

### Study design

WRAP-IT was a multicenter, randomized, single-blinded, interventional clinical trial in patients undergoing a CIED pocket revision, generator replacement or system upgrade, or an initial implantation of a CRT-D (clinicaltrials.gov identifier: NCT02277990). Further details on the trial design, prespecified endpoints, patient inclusion/exclusion criteria, and primary and secondary outcomes have been reported previously.^15^, ^16^, ^17^ The study protocol was approved by the ethics committee at each participating institution and all patients provided written informed consent.

### Definition of hematoma

All adverse events reported as part of the trial were adjudicated by a clinical events committee. Hematomas were identified as adverse events reported in the trial based on the Medical Dictionary for Regulatory Activities (MedDRA® is the international medical terminology developed under the auspices of the International Council for Harmonization) preferred terms, which included the following: implant site hematoma, incision site hematoma, medical device site hematoma. In instances where other preferred terms were indicated, such as implant site bruising and hemorrhage, a physician review committee (KT, BW, FP, PK, MB) assessed the detailed adverse event and invasive intervention description provided by the participating clinical site and reached consensus on whether the event should be reported as a hematoma. This analysis was limited to hematomas occurring in patients within 30 days postprocedure.

### Antithrombotic medication

Use of antithrombotic medication was determined based on case report form (CRF) entries specifying whether the patient was on anticoagulants (AC) and/or antiplatelets (AP), whether they were bridged with heparin, and whether therapy was interrupted. Further details including what specific type of AC was used (ie, warfarin or DOAC) were also collected. If the CRF did not specify warfarin or DOAC use, but did indicate chronic anticoagulant use, the patient was considered to be on warfarin if the international normalized ratio exceeded 1.2 and was considered to be on DOAC if the international normalized ratio was missing or did not exceed 1.2. Interruption of AC was assessed according to details provided on the CRFs. Patients were classified as interrupted if they were taken off warfarin for more than 2 days or off DOAC for more than 1 day or as unknown if not specified in the CRF.

### Statistical analysis

For the purpose of this analysis, patients followed beyond 30 days who had not yet experienced a hematoma were censored at day 30 postprocedure. To evaluate risk factors for hematoma, the Akaike information criterion (AIC), which measures goodness-of-fit through a log-likelihood approach with an added penalty for the number of terms, was used. Minimizing this statistic balances fitting the data while removing variables whose penalty outweighs the improvement in log-likelihood.^18^ A global model of baseline and procedural characteristics was built using stepwise assessment of Cox proportional hazard regression to identify the model minimizing AIC. Since AIC minimization does not depend on *P* values, there is no minimal *P* value restriction. However, *P* values are provided to determine relative confidence among variables remaining in the model. The full list of characteristics included during model selection can be found in Supplemental Table 1.

An initial Cox regression model was developed using all patients that received the intended randomized treatment and included all baseline characteristics and all procedural characteristics that were relevant across all procedures, including de novo procedures. A subsequent Cox regression model was developed that was limited to patients undergoing secondary procedures and excluded patients undergoing de novo procedures and procedural characteristics not relevant to de novo procedures, such as capsulectomy and lead dissection/mobilization. Specific effects of antithrombotic medication types and strategies were assessed with Cox regression models. All analyses were completed using the R statistical package (R Project for Statistical Computing) or SAS software, version 9.4 (SAS Institute, Cary, NC).

## Results

### Patients and procedures

A total of 6800 patients received their intended randomized treatment; the average age was 70.1 years, 71.8% were male, and 85.7% were chronically on AC or AP therapy at the time of their CIED procedure, of whom 50.1% of AC patients had temporary interruption. The baseline characteristics between those patients randomized to the control and envelope groups have been previously reported to be well balanced.^9^^,^^16^ The overall incidence of hematoma in the total cohort was 2.2%, with 151 patients across 76 centers. Summary and full listings of baseline and procedural characteristics for patients that did (n = 151) and did not experience a hematoma (n = 6649) are provided in Table 1 and Supplemental Table 1.

**Table 1:** Baseline and procedure characteristics

Characteristics	Acute hematoma (N = 151)	No hematoma (N = 6649)	Total (N = 6800)
Female	26 (17.2%)	1890 (28.4%)	1916 (28.2%)
Age (years)	72.1 ± 11.5	70.0 ± 12.5	70.1 ± 12.4
BMI (kg/m^2^)	27.4 ± 6.0	29.2 ± 6.2	29.2 ± 6.2
Medical history			
Cardiomyopathy	112 (74.2%)	4521 (68.0%)	4633 (68.1%)
Ischemic	64 (42.4%)	2337 (35.1%)	2401 (35.3%)
Non-Ischemic	48 (31.8%)	2018 (30.4%)	2066 (30.4%)
Hypertrophic	2 (1.3%)	257 (3.9%)	259 (3.8%)
Coronary artery disease	82 (54.3%)	2780 (41.8%)	2862 (42.1%)
Myocardial infarction	47 (31.1%)	1831 (27.5%)	1878 (27.6%)
COPD	26 (17.2%)	828 (12.5%)	854 (12.6%)
Diabetes	41 (27.2%)	2068 (31.1%)	2109 (31.0%)
Renal dysfunction or failure	37 (24.5%)	1069 (16.1%)	1106 (16.3%)
Vascular disease	21 (13.9%)	569 (8.6%)	590 (8.7%)
Stroke	30 (19.9%)	985 (14.8%)	1015 (14.9%)
Cardiovascular surgical history			
CABG	43 (28.5%)	1411 (21.2%)	1454 (21.4%)
Valve surgery	33 (21.9%)	570 (8.6%)	603 (8.9%)
No. of previous CIEDs	1.6 ± 1.3	1.3 ± 1.1	1.3 ± 1.1
Medication use			
Antiplatelets	100 (66.2%)	4009 (60.3%)	4109 (60.4%)
Antibiotics	25 (16.6%)	1120 (16.8%)	1145 (16.8%)
Insulin	15 (9.9%)	775 (11.7%)	790 (11.6%)
Anticoagulants	100 (66.2%)	2868 (43.1%)	2968 (43.6%)
Heart failure/NYHA classifications			
NYHA class I	9 (6.0%)	558 (8.4%)	567 (8.3%)
NYHA class II	42 (27.8%)	1870 (28.1%)	1912 (28.1%)
NYHA class III	34 (22.5%)	1465 (22.0%)	1499 (22.0%)
NYHA class IV	1 (0.7%)	49 (0.7%)	50 (0.7%)
Subject does not have heart failure	29 (19.2%)	1290 (19.4%)	1319 (19.4%)
Class not available	36 (23.8%)	1417 (21.3%)	1453 (21.4%)
Capsulectomy			
None/new system	76 (50.3%)	3991 (60.0%)	4067 (59.8%)
Partial†	65 (43.0%)	2325 (35.0%)	2390 (35.1%)
Complete†	10 (6.6%)	330 (5.0%)	340 (5.0%)
Procedure reason			
Generator replacement w/o lead modification	80 (53.0%)	4191 (63.0%)	4271 (62.8%)
Generator replacement w/ lead modification	13 (8.6%)	438 (6.6%)	451 (6.6%)
Device upgrade (w/ or w/o lead modification)	38 (25.2%)	812 (12.2%)	850 (12.5%)
Pocket or lead revision	2 (1.3%)	67 (1.0%)	69 (1.0%)
Procedure time (hours)	1.1 ± 0.9	0.9 ± 0.8	0.9 ± 0.8
Unconnected leads	35 (23.2%)	894 (13.4%)	929 (13.7%)

Data are reported as n (%) or mean ± SD.

BMI = body mass index; CABG = coronary artery bypass graft; CIED = cardiac implantable electronic device; COPD = chronic obstructive pulmonary disease; NYHA = New York Heart Association; w/ = with; w/o = without.

† For the main analysis model, “partial” and “complete” capsulectomy were combined.

### Main analysis: Multivariable model of hematoma risk (full cohort)

Results for the main multivariable model are shown in Table 2. While all factors included in the AIC selected model contain information worth the added model complexity, the *P* value associated with each factor can be used to describe the level of support for each factor. Factors with a high level of support (*P* < .001) include use of anticoagulants, history of valve surgery, and low body mass index (BMI). Factors with a moderate level of support (.001 < *P* < .05) include antiplatelet use, male sex, history of coronary artery disease, and whether there was an existing pocket at the index procedure (ie, secondary procedure). Factors with a lower level of support but still worth model inclusion (*P* > .05) include history of nonischemic cardiomyopathy, number of previous CIED procedures, procedure time, and whether or not a lead was added, removed, or modified.

**Table 2:** Multivariable model of risk factors for hematoma (full cohort, sorted by hazard ratio)

	N (cat)	Mean (cont)	Hazard ratio	Lower 95% CI	Upper 95% CI	*P* value
Anticoagulant use	2968	NA	2.44	1.69	3.51	<.001
History of valve surgery	603	NA	2.11	1.42	3.15	<.001
Existing pocket reopened	5641	NA	1.92	1.06	3.47	.032
Antiplatelet use	4109	NA	1.66	1.14	2.42	.008
Male	4884	NA	1.63	1.06	2.50	.027
History of coronary artery disease	2862	NA	1.47	1.04	2.10	.031
Lead revised	2529	NA	1.45	0.94	2.23	.090
History of nonischemic cardiomyopathy	2066	NA	1.42	0.99	2.05	.058
Procedure time (hours increase)	NA	0.92	1.21	0.98	1.50	.082
# Previous cardiac device procedures	NA	1.33	1.14	0.99	1.32	.064
BMI (unit decrease)	NA	29.16	1.06	1.03	1.09	<.001

To evaluate risk factors for hematoma, the Akaike information criterion (AIC) was used. Since a lower AIC indicates a better goodness-of-fit/complexity tradeoff, a global model of baseline and procedural characteristics was built using stepwise assessment of Cox proportional hazard regression to identify the model minimizing AIC. Since AIC minimization does not depend on *P* values, there is no minimal *P* value restriction. However, *P* values are provided to determine relative confidence among variables remaining in the model.

BMI = body mass index; cat = categorical variable; CI = confidence interval; cont = continuous variable; NA = not applicable.

All variables listed were associated with an increased risk of hematoma except BMI, which had an inverse relationship with hematoma risk. The highest risk associated with hematoma was anticoagulant use (hazard ratio [HR]: 2.44, 95% confidence interval [CI]: 1.69–3.51, *P* < .001). The other strong associations had similar effects with each unit decrease of BMI (HR: 1.06, 95% CI: 1.03–1.09, *P* < .001) and history of valve surgery (HR: 2.11, 95% CI: 1.42–3.15, *P* < .001). AP use had an additive influence on anticoagulant use (HR: 1.66, 95% CI: 1.14–2.42, *P* = .008).

### Subanalysis: Multivariable model of hematoma risk (secondary procedures)

Since some procedural characteristics were only relevant for secondary procedures, such as whether a capsulectomy was performed, the same AIC minimization modeling strategy was used for only the cohort of patients undergoing a secondary procedure. Following these modifications, a similar set of variables remained in the model, with only capsulectomy entering the model with a lower level of support (*P* = .083) and procedure time exiting the variable list (Supplemental Table 2).

### Antithrombotic use and risk of hematoma

**Figure 1:**
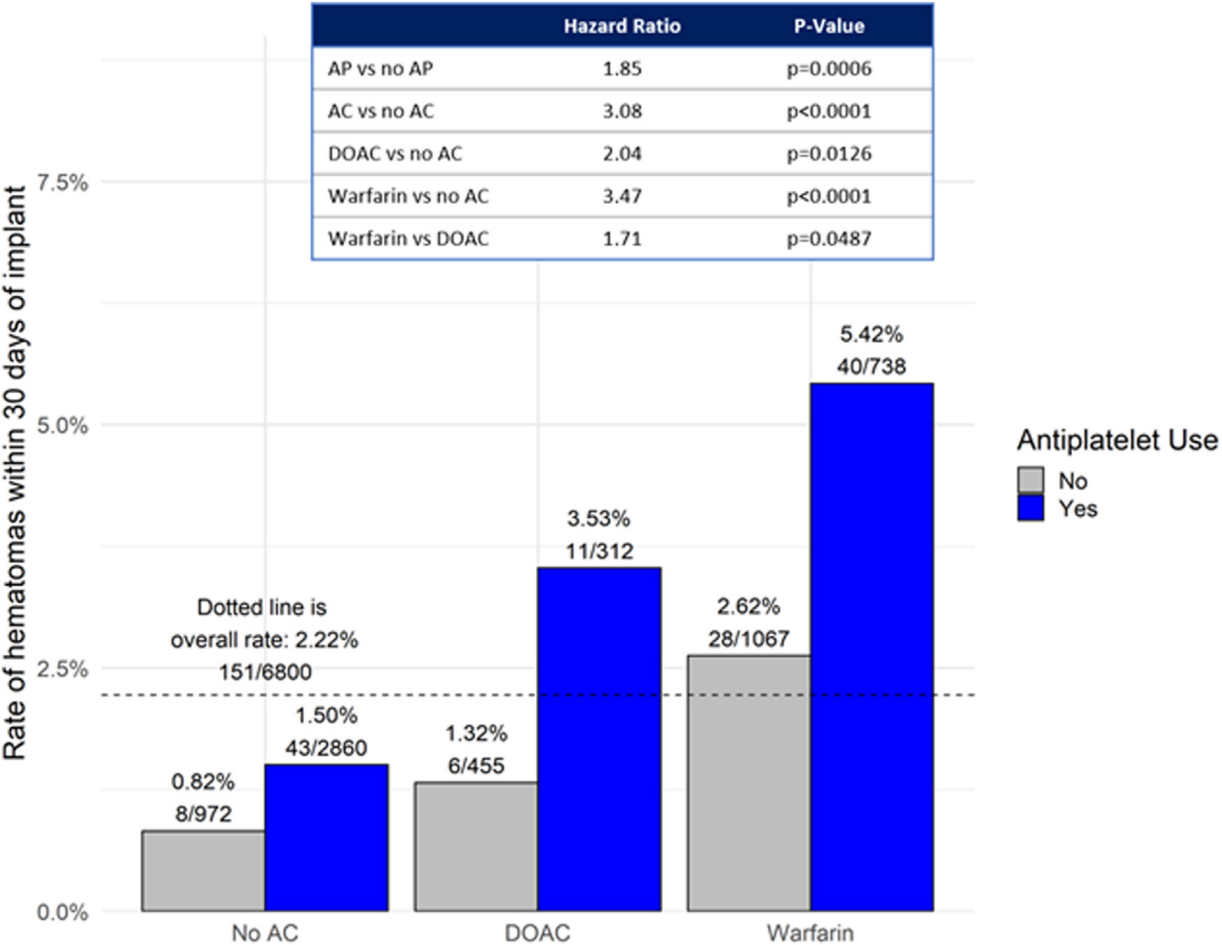
Risk of hematoma stratified by anticoagulant (AC) and antiplatelet (AP) use. Bar chart depicting hematoma rates within 30 days of the patients’ index procedures stratified by antithrombotic use. Hazard ratios (HR), 95% confidence interval (CI), and *P* values are calculated using Cox proportional regression modeling. Hematoma rates varied substantially based on AC use, type (warfarin or direct oral anticoagulant [DOAC]), and with or without use of AP, which was associated with an almost doubling of hematoma risk vs no AP use (HR: 1.85, *P* = .0006) in the general cohort. Among the type of AC used, the risk of hematoma was higher in the warfarin group vs DOAC (HR: 1.71; 95% CI 1.00–2.90; *P* = .0487).

**Figure 2:**
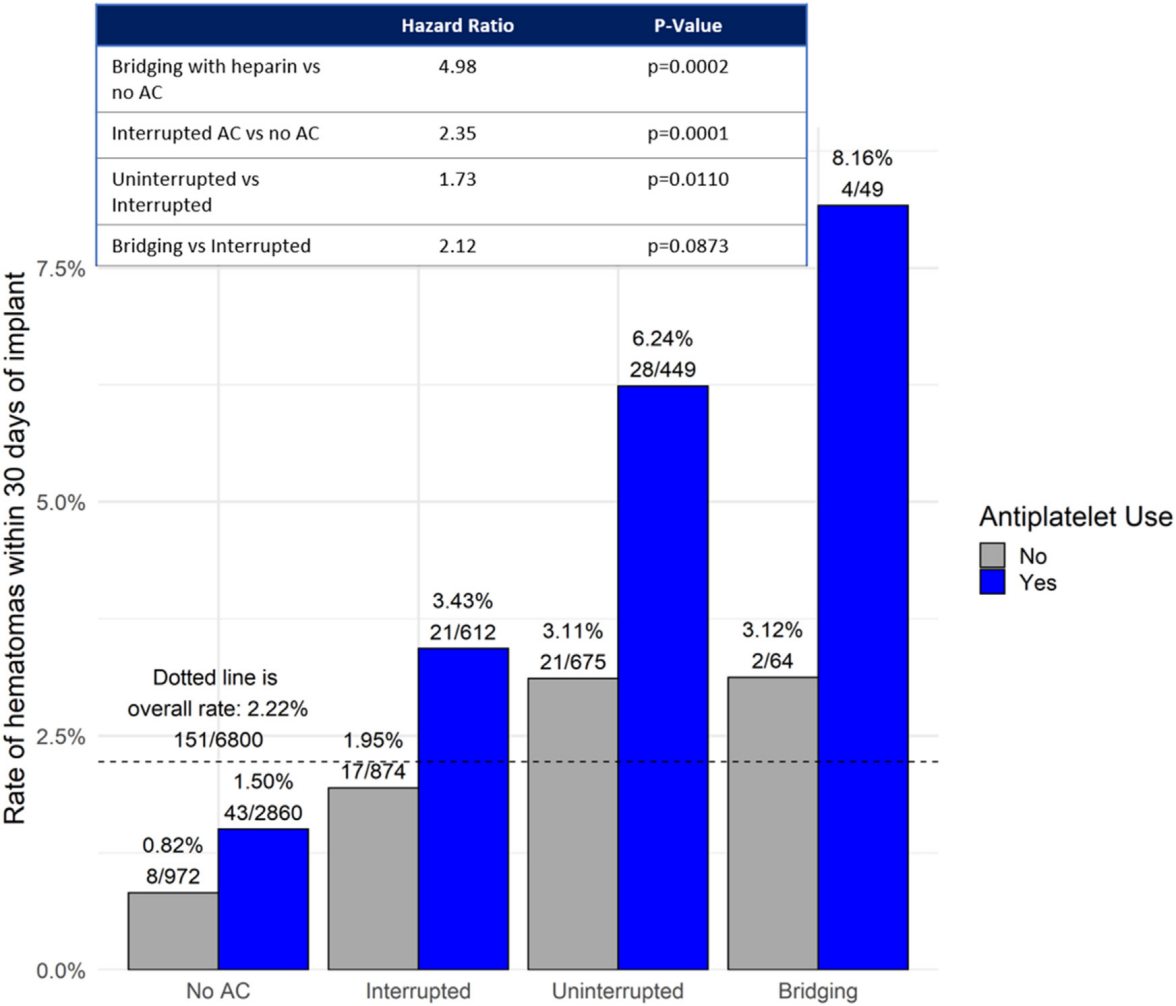
Risk of hematoma stratified by anticoagulant (AC) and antiplatelet (AP) management strategy. Bar chart depicting hematoma rates within 30 days of the patients’ index procedures stratified by antithrombotic strategy. Hazard ratios, 95% confidence intervals, and *P* values are calculated using Cox proportional regression modeling. Bridging with heparin was associated with the highest risk of hematoma when on AP, while an interrupted AC strategy was associated with the lowest risk of hematoma.

Since both the use of AC and AP were highly significant in the multivariable model, risk of hematoma was evaluated in further detail based on the type of AC and the management strategy (interruption vs no interruption, heparin bridging vs no bridging) while also looking at AP therapy. These data are summarized in Table 3. Among the 5828 patients (85.7%) that were on AC or AP therapy at the time of their CIED procedure, 4109 (60.4%) were on AP alone, 2968 (43.6%) were on AC alone, and 1249 (18.4%) were on both AP and AC. Only 972 (14.3%) were on neither. Approximately two-thirds of patients on AC were on warfarin and a quarter were on DOACs. Hematoma rates varied substantially by antithrombotic use, based on type of AC use (warfarin or DOAC), and with or without use of AP, ranging from 0.82% without AC or AP to 2.50% with AC alone to 4.56% with AC and AP (Figure 1). AP use was associated with an almost doubling of hematoma risk vs no AP (HR: 1.85, *P* = .0006) in the general cohort. Among the type of AC used, the risk of hematoma was higher in the warfarin group vs DOAC (HR: 1.71, *P* = .0487). The type of AC management strategy also resulted in varying risk of hematoma (*P* < .0001) compared to no therapy (Figure 2). Bridging with heparin was associated with the highest risk of hematoma (HR: 4.98, *P* = .0002), while an interrupted AC strategy was associated with a significantly lower risk of hematoma (HR: 2.35, *P* = .0001).

**Table 3:** Antithrombotic use at cardiac implantable electronic device procedure

Antithrombotic	No AP (N = 2691 [39.6%])	AP (N = 4109 [60.4%])
No AC	972 (14.3%)	2860 (42.1%)
AC	1719 (25.3%)	1249 (18.4%)
Warfarin	1067 (62.1%)	738 (59.1%)
DOAC	455 (26.5%)	312 (25.0%)
Unknown AC type	197 (11.5%)	199 (15.9%)
AC strategy		
Uninterrupted	675 (39.3%)	449 (35.9%)
Interrupted	874 (50.8%)	612 (49.0%)
Bridging	64 (3.7%)	49 (3.9%)
Unknown AC strategy	106 (6.2%)	139 (11.1%)

Data are reported as n (%).

AC = anticoagulant; AP = antiplatelet; DOAC = direct oral anticoagulant.

## Discussion

In this large prospectively followed cohort of patients undergoing secondary CIED procedures or initial CRT-D implantation, the incidence of hematoma within 30 days is 2.2%, which is within the 1.2%–9.5% range reported in previous studies.^1^, ^2^, ^3^, ^4^, ^5^, ^6^, ^7^ Pocket hematoma is a complication that can render a relatively simple and common procedure, such as a generator change, into a major procedure owing to the potential consequences involved. Clinically significant hematomas associated with CIED procedures can have serious implications, including patient discomfort and worsening quality of life,^7^^,^^19^ need for reoperation to evacuate large hematomas,^20^ prolonged hospitalization,^21^ and cessation of oral anticoagulant therapy and associated risks such as stroke or thrombosis.^20^^,^^22^ Perhaps the biggest negative impact is mainly driven by the well-established correlation between hematoma formation and infection.^23^, ^24^, ^25^, ^26^, ^27^ In prior analysis of the same cohort of WRAP-IT patients, there was an >11-fold increase in infection rate among patients who developed hematomas.^9^ Typically, CIED infection management necessitates device and lead extraction, leading to interruption of device therapy, significant morbidity, and mortality, in addition to major healthcare costs.^28^, ^29^, ^30^

Prior studies have identified potential predictors of hematoma, including patient characteristics, surgical techniques, and the management of antithrombotic therapy,^3^^,^^5^^,^^11^ although findings are inconsistent between reports, and most are single-center studies. To better define and mitigate the risk of hematoma, we performed this analysis to identify risk factors that can be attributed to the development of a hematoma. Our multivariable analysis model identified 11 baseline and procedural characteristics associated with hematoma risk. AC use, lower BMI, history of valve surgery, AP use, prior CIED procedures, and lead revision were associated with increased hematoma risk.

Antithrombotic use, in general, was highly predictive of hematoma risk, and in this global cohort close to 86% of patients were on AC or AP at the time of their CIED procedure. This is not surprising, since the majority of patients undergoing secondary device procedures are older, with multiple comorbidities that render the need for antithrombotics ubiquitous, which further reflects the generalizability of this issue. Varying antithrombotic regimens impacted hematoma risk whereby the use of warfarin was associated with >3× risk, use of DOAC with ∼2× risk, and antiplatelet use with ∼2× risk. AC management strategies had an influence on hematoma risk, with an interrupted AC strategy being associated with the lowest risk of hematoma (HR = 2.35), while heparin bridging (HR = 4.98) and AP use vs no AP use (HR = 1.85) were associated with a higher risk of hematoma. The BRUISE CONTROL study showed that bridging with heparin leads to a higher risk of hematoma formation than with the continuation of warfarin therapy,^7^ while BRUISE CONTROL-2 showed no difference in this risk with the continuation of DOAC vs its interruption.^13^ Our findings confirm those of BRUISE CONTROL when it comes to bridging with heparin; however, interruption of AC (whether warfarin or DOAC) predisposes to significantly lower risk for hematoma formation compared to continuation of AC. These findings highlight the importance of personalized, patient-centric therapy. Many patients are at low-to-moderate risk of thromboembolism and interrupting AC might still be the best strategy to minimize the risk of hematoma, especially in the era of DOAC, which remains to be investigated. The continuation of anticoagulation should only be reserved for those at significantly higher risk, such as those patients with a mechanical valve or recent history of thromboembolism or very high CHA_2_DS_2_-VASc score. Importantly, the risk of thromboembolism should be carefully weighed against the risk of hematoma and its major consequences.

In our cohort, we also observed that the use of AP therapy doubled the risk of hematoma among all patients, whether they were on no AC or whether AP use was concomitant to warfarin or DOAC use. For CIED procedures that are elective in nature, this provides an opportunity to review the patients’ medical regimen. Guidelines for the optimal use of AP therapy, whether for primary prevention or after coronary interventions, continue to evolve, especially among patients on AC for atrial fibrillation.^31^^,^^32^ Perhaps these CIED procedures represent an opportunity to review the antithrombotic regimen of the patient and could trigger a discussion between the electrophysiologist and the interventional or clinical cardiologist to better assess the patient’s needs for these agents, both around the time of the procedure and long term.

### Limitations

Hematoma occurrence was not a prospectively stated objective of the WRAP-IT trial, and as such, data capture was limited to site-specific entries and adverse event descriptions. However, all adverse events and pocket-related complications reported as part of the WRAP-IT trial were adjudicated by a blinded, experienced, independent physician review committee. Second, the management of antithrombotics (AC or AP) or hematomas were not a primary aim of the WRAP-IT trial and as such were not standardized by the trial design but were carried out per the discretion of the physician and/or site-specific practice. The level of procedural detail captured, therefore, does not allow for a granular understanding of the decision to continue or interrupt AC/AP therapy; however, these data are reflective of real-world clinical practice. With regard to data modeling, the variables included in the model were limited to those prospectively collected as part of the WRAP-IT trial. Furthermore, our models would have a preference to select surrogate measures that commonly correlate with multiple characteristics. Finally, a subanalysis including only patients undergoing de novo procedures was limited by the relatively low number of events in this cohort.

## Conclusion

Among WRAP-IT trial patients undergoing CIED generator replacement, system upgrade, or revision or initial CRT-D implantation, the overall risk for hematoma was 2.2% at 30 days postprocedure and 86% of patients were on antithrombotics. Hematoma rates varied substantially based on warfarin (>3× risk) or DOAC use (∼2× risk) and use of antiplatelets, which was associated with an almost doubling of hematoma risk vs no antiplatelet use. Interruption of anticoagulant use was associated with the lowest hematoma risk. The results of this analysis can help inform antithrombotic management, particularly in patients with elevated risk of hematoma.
