# Supplementary material for: Risk factors for hematoma in patients undergoing cardiac device procedures: A WRAP-IT trial analysis
Source: Heart Rhythm O2. 2022 Jun 16;3(5):466–73. doi: 10.1016/j.hroo.2022.05.012 (PMC9626743; doi:10.1016/j.hroo.2022.05.012)
Supplement: Supplemental Table 1-2 [file mmc1.docx]

**SUPPLEMENT**

**Supplemental Table** **1.** **Baseline and Procedure Characteristics (full listing)**

| **Subject Characteristics** | **Acute Hematoma (N = 151)** | **No Hematoma (N = 6649)** | **Total (N = 6800)** |
| --- | --- | --- | --- |
| **Treatment (N, %)** |  |  |  |
| TYRX Envelope | 80 (53.0%) | 3291 (49.5%) | 3371 (49.6%) |
| Control | 71 (47.0%) | 3358 (50.5%) | 3429 (50.4%) |
| **Gender (N, %)** |  |  |  |
| Male | 125 (82.8%) | 4759 (71.6%) | 4884 (71.8%) |
| Female | 26 (17.2%) | 1890 (28.4%) | 1916 (28.2%) |
| **Age (years)** | 72.1 ± 11.5 | 70.0 ± 12.5 | 70.1 ± 12.4 |
| **Body Mass Index** | 27.4 ± 6.0 | 29.2 ± 6.2 | 29.2 ± 6.2 |
| **Systolic Blood Pressure** | 130.7 ± 21.8 | 130.8 ± 20.9 | 130.8 ± 20.9 |
| **Implant Information** |  |  |  |
| **Procedure Reason** |  |  |  |
| Secondary Procedures (not *de novo*) | 133 (88.1%) | 5508 (82.8%) | 5641 (83.0%) |
| Added, removed, or modified a lead | 71 (47.0%) | 2458 (37.0%) | 2529 (37.2%) |
| **Tissue Layer** |  |  |  |
| Tissue Layer unknown | 0 (0.0%) | 30 (0.5%) | 30 (0.4%) |
| Subcutaneous | 143 (94.7%) | 6191 (93.1%) | 6334 (93.1%) |
| Not Subcutaneous | 8 (5.3%) | 428 (6.4%) | 436 (6.4%) |
| **Electrocautery Type** |  |  |  |
| None | 6 (4.0%) | 556 (8.4%) | 562 (8.3%) |
| Standard Electrocautery | 97 (64.2%) | 4174 (62.8%) | 4271 (62.8%) |
| Low Temp Electrocautery | 48 (31.8%) | 1917 (28.8%) | 1965 (28.9%) |
| **Procedure Information** |  |  |  |
| Hemostatic Solution Used | 16 (10.6%) | 455 (6.8%) | 471 (6.9%) |
| Hemostatic Pocket Wash Used | 8 (5.3%) | 260 (3.9%) | 268 (3.9%) |
| **Number of Active Leads** |  |  |  |
| One active lead | 14 (9.3%) | 760 (11.4%) | 774 (11.4%) |
| Two active leads | 60 (39.7%) | 2584 (38.9%) | 2644 (38.9%) |
| Three active leads | 71 (47.0%) | 3195 (48.1%) | 3266 (48.0%) |
| Four active leads | 6 (4.0%) | 110 (1.7%) | 116 (1.7%) |
| **Closure modes** |  |  |  |
| Sutures | 149 (98.7%) | 6469 (97.3%) | 6618 (97.3%) |
| Staples | 6 (4.0%) | 334 (5.0%) | 340 (5.0%) |
| Dermabond | 31 (20.5%) | 1217 (18.3%) | 1248 (18.4%) |
| Steristrips | 14 (9.3%) | 539 (8.1%) | 553 (8.1%) |
| **Procedure Time (hours)** | 1.1 ± 0.9 | 0.9 ± 0.8 | 0.9 ± 0.8 |
| **Medical History** |  |  |  |
| Ischemic Cardiomyopathy | 64 (42.4%) | 2337 (35.1%) | 2401 (35.3%) |
| Non-Ischemic Cardiomyopathy | 48 (31.8%) | 2018 (30.4%) | 2066 (30.4%) |
| Hypertrophic Cardiomyopathy | 2 (1.3%) | 257 (3.9%) | 259 (3.8%) |
| Coronary artery disease | 82 (54.3%) | 2780 (41.8%) | 2862 (42.1%) |
| Idiopathic Structural Heart Disease | 4 (2.6%) | 95 (1.4%) | 99 (1.5%) |
| Idiopathic Electrical Disease | 2 (1.3%) | 218 (3.3%) | 220 (3.2%) |
| Left Ventricular Hypertrophy | 5 (3.3%) | 306 (4.6%) | 311 (4.6%) |
| COPD | 26 (17.2%) | 828 (12.5%) | 854 (12.6%) |
| Diabetes, Insulin Dependent | 13 (8.6%) | 670 (10.1%) | 683 (10.0%) |
| Diabetes, Non-Insulin Dependent | 28 (18.5%) | 1398 (21.0%) | 1426 (21.0%) |
| Renal dysfunction or failure | 37 (24.5%) | 1069 (16.1%) | 1106 (16.3%) |
| Vascular disease | 21 (13.9%) | 569 (8.6%) | 590 (8.7%) |
| Ablation | 22 (14.6%) | 786 (11.8%) | 808 (11.9%) |
| Stroke or TIA or Embolism | 30 (19.9%) | 985 (14.8%) | 1015 (14.9%) |
| Myocardial infarction | 47 (31.1%) | 1831 (27.5%) | 1878 (27.6%) |
| Hypertension | 101 (66.9%) | 4360 (65.6%) | 4461 (65.6%) |
| Hyperlipidemia | 94 (62.3%) | 3886 (58.4%) | 3980 (58.5%) |
| Cardiac Arrest | 7 (4.6%) | 387 (5.8%) | 394 (5.8%) |
| Cancer | 16 (10.6%) | 564 (8.5%) | 580 (8.5%) |
| Liver Disease | 2 (1.3%) | 88 (1.3%) | 90 (1.3%) |
| History of Smoking | 78 (51.7%) | 2878 (43.3%) | 2956 (43.5%) |
| **Cardiovascular Surgical History** |  |  |  |
| CABG | 43 (28.5%) | 1411 (21.2%) | 1454 (21.4%) |
| Valve surgery | 33 (21.9%) | 570 (8.6%) | 603 (8.9%) |
| **Number of Previous CIEDs** | 1.6 ± 1.3 | 1.3 ± 1.1 | 1.3 ± 1.1 |
| **Atrial Arrhythmias (N, %)** |  |  |  |
| Any Atrial Arrhythmia | 99 (65.6%) | 3200 (48.1%) | 3299 (48.5%) |
| Left Bundle Branch Block | 34 (22.5%) | 1897 (28.5%) | 1931 (28.4%) |
| **Medication Use** |  |  |  |
| Antiplatelets | 100 (66.2%) | 4009 (60.3%) | 4109 (60.4%) |
| Antibiotics | 25 (16.6%) | 1120 (16.8%) | 1145 (16.8%) |
| Insulin | 15 (9.9%) | 775 (11.7%) | 790 (11.6%) |
| Anticoagulants | 100 (66.2%) | 2868 (43.1%) | 2968 (43.6%) |
| **CIED type received** |  |  |  |
| **Low Power** |  |  |  |
| Pacemaker | 23 (15.2%) | 1371 (20.6%) | 1394 (20.5%) |
| CRT-P | 11 (7.3%) | 282 (4.2%) | 293 (4.3%) |
| **High Power** |  |  |  |
| ICD | 35 (23.2%) | 1726 (26.0%) | 1761 (25.9%) |
| CRT-D | 82 (54.3%) | 3270 (49.2%) | 3352 (49.3%) |
| **Heart Failure/NYHA Classifications (N, %)** |  |  |  |
| NYHA Class I | 9 (6.0%) | 558 (8.4%) | 567 (8.3%) |
| NYHA Class II | 42 (27.8%) | 1870 (28.1%) | 1912 (28.1%) |
| NYHA Class III | 34 (22.5%) | 1465 (22.0%) | 1499 (22.0%) |
| NYHA Class IV | 1 (0.7%) | 49 (0.7%) | 50 (0.7%) |
| Subject does not have heart failure | 29 (19.2%) | 1290 (19.4%) | 1319 (19.4%) |
| Class not available | 36 (23.8%) | 1417 (21.3%) | 1453 (21.4%) |
| Data are reported as n (%) or mean ± SD  BMI = body mass index; CABG = coronary artery bypass graft; CIED = cardiac implantable electronic device; COPD = chronic obstructive pulmonary disease; CRT-D = cardiac resynchronization therapy–defibrillator; CRT-P = cardiac resynchronization therapy–pacemaker; ICD = implantable cardioverter-defibrillator; NYHA = New York Heart Association, TIA = transient ischemic attack  *Patients with CIED infection 12 months before trial enrollment | | | |
|  | | | |

**Supplemental Table 2. Multivariable Model of Risk factors for Hematoma (secondary procedures cohort)**

|  | **N (Cat)** | **Mean (cont)** | **Hazard Ratio** | **Lower 95% CI** | **Upper 95% CI** | **p-Value** |
| --- | --- | --- | --- | --- | --- | --- |
| History of Valve Surgery | 506 | NA | 2.55 | 1.70 | 3.84 | <0.001 |
| Anticoagulant Use | 2541 | NA | 2.37 | 1.60 | 3.52 | <0.001 |
| BMI (Unit Decrease) | NA | 29.10 | 1.06 | 1.03 | 1.10 | <0.001 |
| Lead revised | 1370 | NA | 1.75 | 1.23 | 2.49 | 0.002 |
| History of Coronary Artery Disease | 2362 | NA | 1.59 | 1.09 | 2.31 | 0.016 |
| History of non-ischemic cardiomyopathy | 1509 | NA | 1.51 | 1.02 | 2.23 | 0.039 |
| Male | 4017 | NA | 1.59 | 1.01 | 2.50 | 0.043 |
| Number of Previous Cardiac Device Procedures | NA | 1.61 | 1.15 | 1.00 | 1.32 | 0.046 |
| Antiplatelet Use | 3347 | NA | 1.48 | 1.00 | 2.20 | 0.050 |
| Capsulectomy | 2730 | NA | 1.36 | 0.96 | 1.91 | 0.083 |
